# Identifying functional modules in interaction networks through overlapping Markov clustering

**DOI:** 10.1093/bioinformatics/bts370

**Published:** 2012-09-03

**Authors:** Yu-Keng Shih, Srinivasan Parthasarathy

**Affiliations:** Department of Computer Science and Engineering, the Ohio State University, Columbus 43210-1277, OH USA.

## Abstract

**Motivation:** In recent years, Markov clustering (MCL) has emerged as an effective algorithm for clustering biological networks—for instance clustering protein–protein interaction (PPI) networks to identify functional modules. However, a limitation of MCL and its variants (e.g. regularized MCL) is that it only supports hard clustering often leading to an impedance mismatch given that there is often a significant overlap of proteins across functional modules.

**Results:** In this article, we seek to redress this limitation. We propose a soft variation of Regularized MCL (R-MCL) based on the idea of iteratively (re-)executing R-MCL while ensuring that multiple executions do not always converge to the same clustering result thus allowing for highly overlapped clusters. The resulting algorithm, denoted soft regularized Markov clustering, is shown to outperform a range of extant state-of-the-art approaches in terms of accuracy of identifying functional modules on three real PPI networks.

**Availability:** All data and codes are freely available upon request.

**Contact:** srini@cse.ohio-state.edu

**Supplementary Information:** Supplementary data are available at *Bioinformatics* online.

## 1 INTRODUCTION

Advances in technology have enabled scientists to determine, identify and validate pairwise protein interactions through a range of experimental approaches. Recently, several high-throughput approaches have produced a large scale of protein–protein interaction (PPI) datasets. These approaches include yeast two-hybrid, protein co-immunoprecipitation followed by mass spectrometry (MS), protein chip technologies and tandem affinity purification (TAP) with MS. Such data have led researchers to discover protein functions through PPI networks, in which a node represents a protein and an edge mimics an interaction between two proteins. A fundamental goal here is to discover functional modules or protein complexes in order to predict the function of unannotated proteins.

The fundamental concept of identifying functional modules is that a pair of proteins interacting with each other has higher probability of sharing the same function than two proteins not interacting with each other. The dense sub-networks in a PPI network can therefore be identified as functional modules. Thus, identifying functional modules is similar to detecting communities (clusters) in a network (graph). However, traditional community detection algorithms are usually ‘hard’ clustering algorithms, i.e. they produces non-overlapped clusters, whereas functional modules are highly ‘overlapped’ (Li *et al.*, 2010). As a result, a number of ‘soft’ clustering algorithms have been recently proposed to identify functional modules in a PPI network, and they can be grouped into three categories.

The first category includes algorithms such as Peacock (Gregory, 2009), hub-duplication (Ucar *et al.*, 2006) and DECAFF (Li *et al.*, 2007). These algorithms identify the bridge nodes at the beginning, i.e. nodes belong to multiple clusters, and then either duplicate or remove the bridge nodes from the network. A hard clustering algorithm is then applied on the modified network. The problem with this approach is that only the identified bridge nodes can belong to multiple clusters, and it is conflicted with the literature (Ashburner *et al.*, 2000) that a large fraction of proteins belong to multiple functional modules. For example, in the yeast network in BioGRID database (Stark *et al.*, 2011), there are 3085 proteins annotated by low-level Gene Ontology (GO) terms, whose information content (see Sec 4.1) is higher than 2.5, and 2392 of 3085 proteins are annotated by at least two of these GO terms.

Algorithms in the second category adopt line-graph transformation. These algorithms (Ahn *et al.*, 2010; PereiraLeal *et al.*, 2004) first transform the input network into a line graph, in which a node represents an edge in the original network. Then, a hard clustering algorithm is applied on the line graph, so edges are clustered instead of nodes. A node in the original network belongs to multiple clusters if its incident edges are clustered into different clusters. It has been pointed out in the literature (Fortunato, 2010) that clustering edges has a similar issue as clustering nodes: a ‘bridge edge’ that connects nodes of different clusters can only be clustered into one cluster by the line-graph technique. Furthermore, while functional modules are so highly overlapped that an interaction might belong to multiple modules, these algorithms cannot successfully identify all overlapped functional modules.

Algorithms in the third category aim to find local dense subnetworks instead of globally clustering a graph. Each node forms a singleton cluster at the beginning, and then each cluster iteratively adds a neighbor node according to different criteria. Algorithms in this category include MCODE (Bader and Hogue, 2003), CFinder (Adamcsek *et al.*, 2006), DPClus (Altaf-Ul-Amin *et al.*, 2006), IPCA (Li *et al.*, 2008), MoNet (Luo *et al.*, 2007), CORE (Leung *et al.*, 2009), COACH (Wu *et al.*, 2009), DME (Georgii *et al.*, 2009), RRW (Macropol *et al.*, 2009), NWE (Maruyama and Chihara, 2011), SPICi (Jiang and Singh, 2010), HUNTER (Chin *et al.*, 2010) and HC-PIN (Wang *et al.*, 2011).

Although the resulting clusters could be highly overlapped, one main drawback of those algorithms is that the criterion for adding a node usually considers relatively local topology. Given that PPI networks are estimated to be quite noisy (Brohee and Helden, 2006), these algorithms could add several nodes connected by noisy edges.

In addition to those in the above three categories, there are some other algorithms, such as RNSC (King *et al.*, 2004), principal component analysis (PCA)-based consensus clustering (Asur *et al.*, 2007) and Markov clustering (MCL) algorithm (Dongen, 2000), which have targeted identification of functional modules. The detail of most above-mentioned algorithms can be found in recent surveys (Fortunato, 2010; Li *et al.*, 2010). MCL, which is based on manipulation of transition probabilities or stochastic flows between nodes of the graph, is shown to be particularly noise-tolerant as well as effective in identifying high-quality functional modules (Brohee and Helden, 2006; Vlasblom and Wodak, 2009). Several studies, such as (Friedel *et al.*, 2009), (Moschopoulos *et al.*, 2008) and (Srihari *et al.*, 2009), have adopted MCL as a base algorithm to produce more accurate results. Recently, (Satuluri *et al.*, 2010) propose an efficient and robust variation of MCL, called Regularized MCL (R-MCL). They show that R-MCL's regularize operation and balance parameter can improve the accuracy of identifying functional modules. Nevertheless, MCL and R-MCL only generate non-overlapped clusters, and they always assign all proteins into clusters while not all proteins are functionally annotated. As a result, MCL and R-MCL usually produce more false-positive clusters than other algorithms (Brohee and Helden, 2006; Li *et al.*, 2010).

In this article, we redress the limitation of R-MCL and propose a new variation called ‘Soft’ R-MCL (SR-MCL), which produces overlapped clusters. The intuition of SR-MCL is to produce overlapped clusters by iteratively re-executing R-MCL while ensuring the resulting clusters are not always the same. In order to produce different clusterings in each iteration, the stochastic flows are penalized if they flow into a node that was an attractor node in previous iterations. Since iteratively re-executing R-MCL would produce several redundant and low-quality clusters, a postprocessing is applied to remove those clusters. Only a cluster that is not removed by the post-processing is predicted as a functional module, so not all proteins are assigned into clusters.

We have conducted a series of experiments on three networks in *Saccharomyces cerevisiae*. Based on the gold standard annotation, GO terms (Ashburner *et al.*, 2000), we find that SR-MCL has significantly higher accuracy than R-MCL. SR-MCL also outperforms a range of algorithms on these three networks. Since it has been pointed out that there are different scales of potential functional relevance within a PPI network (Lewis *et al.*, 2010), we also demonstrate that R-MCL is capable of identifying both the parent module as well as the child module in the GO hierarchy.

## 2 TERMINOLOGY

Let *G* = (*V, E*) denote a PPI network, which is an undirected graph excluding self-loops, where *V* is the set of nodes (proteins), *E* is the set of edges (interactions) and *n* = |*V* |. Each edge is denoted by (*v_i_*,*v_j_*), *v_i_*,*v_j_* ∈ *V*. *w*((*v_i_*,*v_j_*)) is the weight of an edge (*v_i_*,*v_j_*), which represents the confidence level of the interaction in a weighted PPI network. If the network is unweighted, the weight of an edge is always 1. Let *A* be the adjacency matrix of the graph such that

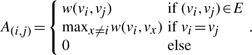

A column stochastic matrix M is a *n* by *n* matrix that can be interpreted as the matrix of the transition probabilities of a random walk (or a Markov chain) defined on the graph. Specifically, *M*_(*i,j*)_ represents the probability of a transition from *v_j_* to *v_i_*. We also refer to the transition probability from *v_i_* to *v_j_* as the flow from *v_i_* to *vj*. The canonical flow matrix *M*_G_ is defined as 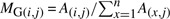.

## 3 METHOD

### 3.1 Prior work on Markov clustering

MCL and R-MCL are graph clustering algorithms based on a simulation of stochastic flows on the graph. MCL consists of two operations on a stochastic matrix: ‘Expand’ and ‘Inflate.’ The Expand operation is simply *M* = *M* × *M*, and the Inflate operation raises each entry in the matrix M to the inflation parameter *r* (*r >* 1, and typically set to 2) followed by re-normalizing the sum of each column to 1. These two operations are applied in alternation iteratively, starting with *M* = *M*_G_, where *M*_G_ is the canonical flow matrix. In R-MCL, Expand is replaced by ‘Regularize’, which is *M* = *M* × *M*_G_. The Expand and Regularize operations spread the flow out of a vertex to potentially new nodes. This has the effect of enhancing within-cluster flows as there are more paths between two nodes that are in the same cluster than between those in different clusters. At the start of this process, the distribution of flows out of a node is relatively smooth and uniform; as more iterations are executed, all the nodes within a tightly linked group of nodes will start to flow to one node within the group. This allows us to identify all the nodes that flow to the same ‘attractor node’ as belonging to one cluster. (Satuluri *et al.*, 2010) additionally introduce a balance parameter into R-MCL. For a complete description of MCL and R-MCL, the reader is referred elsewhere (Dongen, 2000; Satuluri and Parthasarathy, 2009; Satuluri *et al.*, 2010).

### 3.2 Overlapping MCL

**Fig. 1.**
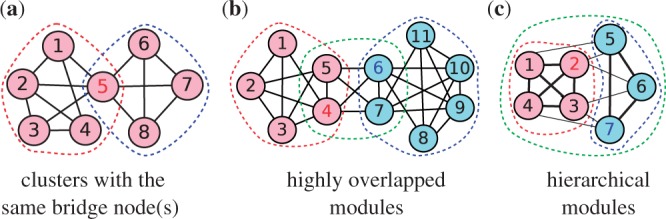
Three toy examples pointing out the problems of R-MCL. All edges have the weight 1 except that the thin edges in (c) have weight 0.5. The color of the nodes represents the result of R-MCL, and the red/blue numbers indicate the attractor nodes w.r.t. the red/blue clusters. The dash circles indicate functional modules

Although R-MCL is effective and efficient in hard clustering, it has three issues in identifying functional modules, which are usually hierarchical and highly overlapped. First, R-MCL usually merges functional modules sharing the same (bridge) node(s). A bridge node usually interacts with a large number of nodes in a PPI network, so it is likely to become an attractor node. As shown in Figure 1a, two modules are clustered together by R-MCL with the bridge node *v*_5_ being the attractor node. Second, R-MCL cannot identify modules with large overlaps since it is a hard clustering algorithm. For example, R-MCL cannot produce any cluster similar to the green module in Figure 1b. Third, again, because R-MCL is a hard clustering algorithm, R-MCL is unable to identify hierarchical modules. As shown in Figure 1c, R-MCL only produces two clusters matching the two children (blue and red) modules, while no cluster can match the parent (green) module.

In order to overcome these three issues, we propose a variation, SR-MCL. The intuition of SR-MCL is to iteratively re-execute R-MCL while ensuring the clusters produced are not always the same. Thus, the resulting clusters can be overlapped if clusters produced in all iterations are incorporated. The clustering produced in each iteration is differentiated by ‘penalizing’ the attractor nodes, i.e. decreasing the flow to a node that has been an attractor node in previous iterations. Specifically, the penalty is introduced as follows: in the Inflate operation, the flow to a node that has been an attractor node *x* times in previous iterations is raised to *r* × *β^x^*, where *β >* 1 is the user-specified penalty ratio. Therefore, if a node has not been an attractor node, the flow to it is still raised to the inflation parameter *r*, and more times a node has been an attractor node, more severe penalty is introduced to it. The penalty results in possibly different attractor nodes and therefore possibly different clusters. Moreover, since the bridge node is likely to be the attractor in R-MCL, by penalizing attractor nodes, we can correctly produce clusters sharing the same bridge node. For example, in Figure 1a, R-MCL only identifies one cluster in the first iteration, but in the second iteration, the attractor node *v*_5_ is penalized so SR-MCL could produce two clusters matching the two modules.


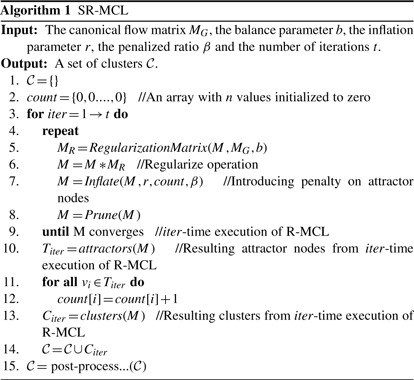


The procedure of SR-MCL is shown in Algorithm 1. Line 4 to Line 9 are the same as R-MCL except introducing the penalty ratio *β* [the detail of R-MCL, including the usage of parameters *r* and *b*, is illustrated elsewhere (Satuluri *et al.*, 2010)]. In the Inflation operation, each entry *M_ij_* in the matrix *M* is raised to *r* × *β*^count[^*^i^*^]^, and then the sum of each column is re-normalized to 1. Count[*i*] is the number of times that *v_i_* has been an attractor node, and *t* is the number of times that R-MCL is executed. Since R-MCL is very efficient (only takes less than 1 s in a modern dual-core machine) in clustering a PPI network, which typically contains less than 10 000 nodes and 100 000 edges, and the difference between clusterings produced by each iteration should be so slight that every possible cluster is produced, we suggest that *t* is set to a large number from 10 to 50 and *β* is set to a relative small number (1.25 in default). Although this setting would result in several redundant clusters, the post-processing, which will be introduced in the next section, can filter out those clusters.

### 3.3 Post-processing

As the resulting clustering from iterative execution of R-MCL could contain several redundant and low-quality clusters, those clusters should be removed. The pseudo code of our post-processing is shown in Algorithm 2. First, we use one of three simple quality functions, ‘density, clustering coefficient’, and density multiply by the square root of size (denoted by density × sqrt(size)), to evaluate the quality of each cluster. The reason of using density × sqrt(size) is that a PPI network is usually relatively sparse, so simply adopting density as the quality function might result in a huge number of too small clusters. Note that other quality functions, such as those discussed by (Lewis *et al.*, 2010), can also be applied. Here, we aim to show that a simple quality function can make SR-MCL produce clusters accurately matching functional modules. The chosen quality function is denoted by *qf*. We remove all clusters whose *qf* value is below a user-specified threshold *ω*. The value of *ω* depends on *qf* and the network's property. Furthermore, all clusters whose size is ≤2 are also removed.


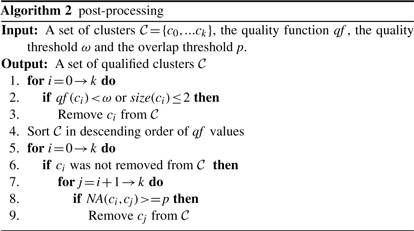


After removing low-quality clusters, we examine whether each cluster is redundant or not in the descending order of its *qf* value. A cluster *c_j_* is removed if there exists a cluster *c_i_* that *qf* (*c_i_*) *>*= *qf* (*c_j_*) and NA(*c_i_*,*c_j_*) *> p*, where *p* is another user-specified threshold and NA is neighborhood affinity (Bader and Hogue, 2003):
(1)
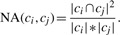

Thus, *p* is used to control the degree of overlap among clusters. The higher *p* produces higher overlapped clusters and vice versa. As the functional modules are highly overlapped, we suggest that *p* is set from 0.3 to 0.8. If *ω* becomes larger and *p* is decreased, the post-processing removes more clusters, so the remaining high-quality clusters can precisely match functional modules, but more functional modules could not be identified. On the other hand, less *ω* and larger *p* result in more clusters, so the resulting clusters can identify more functional modules; however, the result contains relatively redundant and low-quality clusters, so a number of resulting clusters cannot precisely match functional modules.

## 4 RESULTS

### 4.1 Datasets and metrics

We report results on three PPI networks of *S. cerevisiae* extracted from DIP (version October 27, 2011) (Salwinski *et al.*, 2004), BioGRID (version 3.1.81) (Stark *et al.*, 2011) and WI-PHI (Kiemer *et al.*, 2007), respectively. DIP and BioGRID are unweighted networks and WI-PHI is a weighted network. The weight of WI-PHI is adjusted by min-max normalization. All self-loops are removed, and for BioGRID, we only extracted low-throughput experiments that were used since these interactions have higher precision (Paccanaro *et al.*, 2005). In order to evaluate the functional modules identification result, we used GO (Ashburner *et al.*, 2000) as the gold standard clusters for ground truth validation. GO terms is a set of hierarchical annotations, in which low-level (general) terms contain most proteins in a network. As most functional modules identification algorithms produce small clusters which can be identified as high-level (specific) GO terms, we aim to evaluate the results only based on high-level GO terms. Therefore, for each network, we only use the GO terms whose information content (IC) is higher than 2. The information content of a GO term *g* is defined as IC(*g*)= −log(|*g*|*/*|root|), where root is the corresponding GO category (biological process, molecular function or cellular component) of *g*. We moreover remove GO terms annotating 2 or less proteins. The detail of these three networks are shown in Table 1. Except comparing with existing algorithms that can only be applied on unweighted networks, we mainly show the results on WI-PHI, since the edge weight is useful for all weighted network clustering algorithms, including MCL and R-MCL.

**Table 1.** Information of the three yeast networks used in the experiment

Name	|*V* |	|*E*|	|*V* ∈ *GO*|	*avg*(*GO*)	|*GO*|
BioGRID	4364	25464	3771	10.73	3033
DIP	4995	21875	3822	11.30	3038
WI-PHI	5953	49607	4338	12.19	3262

|*V* ∈ *GO*| is the number of proteins annotated by any GO term we used; *avg*(*GO*) is the average GO term size and |*GO*| is the number of GO terms.

We adopt the widely used metric *F*-measure (Li *et al.*, 2010) to evaluate the accuracy of clusters produced. *F*-measure can evaluate not only the accuracy of the clusters matching functional modules but also the accuracy of functional modules matching the clusters. Given a clustering result 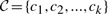, in which singleton clusters are removed and the gold standard clusters (e.g. GO terms) 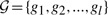, *F*-measure, based on neighborhood affinity (1), is the harmonic mean of precision and recall, which are defined as
(2)
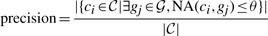

(3)
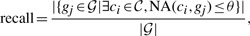

where *θ* is set to a typically value 0.25. We moreover propose a new version of *F*-measure which does not require the threshold *θ*. The equations of new precision and new recall are shown in Supplementary. (The performance of these new metrics is shown in Supplementary.)

The usage and the suggested range of each parameter are listed in Supplementary Table 1. Generally, only *r*, *p* and *ω* need to be tuned and the parameter tuning is straightforward. The parameters (*r*, *b*, *t*, *β*, *p*) of SR-MCL were set to default values (2.0, 0.5, 30, 1.25, 0.6), respectively, in all experiments unless otherwise noted.

### 4.2 The choice of quality function

**Fig. 2.**
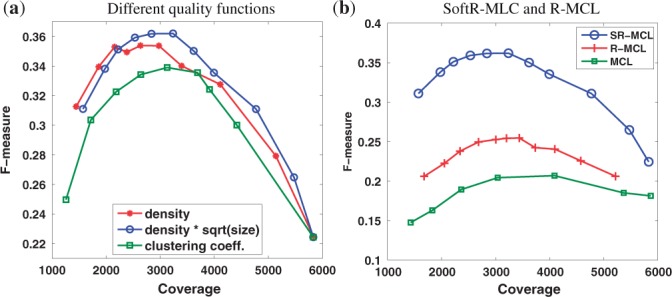
The comparison of F-measure on WI-PHI

We compared the three quality functions mentioned in Section 3.3: density, clustering coefficient and density × sqrt(size). For each quality function, we varied the quality threshold *ω* in order to yield different clusterings in various ‘coverages’, which are the numbers of nodes assigned to any cluster. The result is shown in Figure 2a. Since a too large coverage yields very small precision, and a too small coverage yields very small recall, all quality functions obtain maximal *F*-measure when the coverage is ~2000 to 3500. It is clear that clustering coefficient produces the worst result and density × sqrt(size) produces the best. As mentioned in Section 3.3, purely using density along has slightly lower *F*-measure as it is biased towards small clusters, since the PPI networks are generally sparse. For simplicity, in the following experiments, we use density × sqrt(size) as the quality function.

### 4.3 Comparison with MCL and R-MCL

In this section, we report the benefit of iterative executing R-MCL with penalty on attractor nodes. We use density × sqrt(size) as the quality function to prune the results of MCL, R-MCL, and SR-MCL. The parameters *r* of MCL and (*r*, *b*) of R-MCL are set to the same values as SR-MCL. Again, we varied *ω* to yield different clusterings with various coverages. As can be seen in Figure 2b, SR-MCL always yields significantly higher *F*-measure than R-MCL, while R-MCL's F-measure is higher than MCL's. Drilling down, we observe that the improvement primarily stems from corresponding improvement of recall and to a lesser extent precision (see Supplementary Figs. 1 and S2). This demonstrates that the clusters produced by SR-MCL can more accurately match functional modules as attractor nodes are penalized.

### 4.4 Comparison with state-of-the-art algorithms

We compare SR-MCL with MCL (Dongen, 2000), MCODE (Bader and Hogue, 2003), RNSC (King *et al.*, 2004), CFinder (Adamcsek *et al.*, 2006), DPClus (Altaf-Ul-Amin *et al.*, 2006), IPCA (Li *et al.*, 2008), CORE (Leung *et al.*, 2009), COACH (Wu *et al.*, 2009), RRW (Macropol *et al.*, 2009), HUNTER (Chin *et al.*, 2010), R-MCL (Satuluri *et al.*, 2010), SPICi (Jiang and Singh, 2010), Link Community (LinkCom) (Ahn *et al.*, 2010) and NWE (Maruyama and Chihara, 2011). RRW, NWE, HUNTER and SPICi are designed for weighted networks, so we compared SR-MCL with them on WI-PHI. LinkCom can be applied on both unweighted and weighted network, so all three networks are used. The rest of these algorithms can only be applied on unweighted networks, so they are compared with SR-MCL on DIP and BioGRID. We tuned each algorithm to its best parameter setting but generally found that the default parameter setting generates the best results. The values of each algorithm's parameters are reported in Supplementary Table S2. For R-MCL and MCL, here we show the results without using any quality function to prune out their results. For SR-MCL, we set *p* to 0.8 in this experiment, and we choose 0.4 and 1.2 as *ω* for WI-PHI and BioGRID/DIP, respectively, since these parameters generally yield the best *F*-measure. Finally, we prune out all clusters whose size is less than or equal to 2 in all algorithms' clustering results.

The information of all clustering results is reported in Tables 2 and 3. Since other algorithms generally produce smaller clusters than SR-MCL with above parameter setting, we additionally evaluated the results based on a set of smaller GO terms GO_spec_, which contains GO terms whose information content is higher than 2.5. Detailed information can be found in Supplementary Table S3. For *GO*_spec_, we changed the inflation parameter *r* of SR-MCL to 4.5, which results in smaller clusters. For example, in BioGRID, the average cluster size is reduced from 9.59 to 7.23 if *r* is increased from 2.0 to 4.5.

**Table 2.** The information of clusters produced by SR-MCL and other weighted network clustering algorithms on WI-PHI. avg(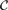) is the average size of clusters

Algorithm	RRW	NWE	HUNTER	SPICi	LinkCom	MCL	R-MCL	SR-MCL
# clusters	1014	442	46	127	4219	649	897	1828
avg( 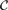 )	6.22	5.85	34.80	7.82	4.93	8.33	5.82	9.54
coverage	3523	2134	1370	994	3467	5407	5217	3118

**Table 3.** The information of clusters produced by SR-MCL and other unweighted network clustering algorithms on BioGRID and DIP

	Algorithm	CFinder	COACH	CORE	DPClus	IPCA	MCODE	RNSC	LinkCom	MCL	R-MCL	SR-MCL
BioGRID	# clusters	816	1248	615	591	1526	81	579	3160	475	559	2611
	avg( 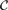 )	6.57	9.37	7.38	6.54	8.79	14.89	4.51	3.71	8.08	7.18	9.59
	coverage	2959	2764	2696	2976	2414	1206	2615	3048	3838	4018	3381
DIP	# clusters	609	903	772	827	989	63	549	949	623	848	1038
	avg( 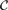 )	6.18	8.90	5.30	5.28	8.80	19.00	3.89	4.00	6.57	5.25	9.84
	coverage	2135	1999	2471	3258	1525	1032	2133	1412	4096	4456	2047

**Fig. 3.**
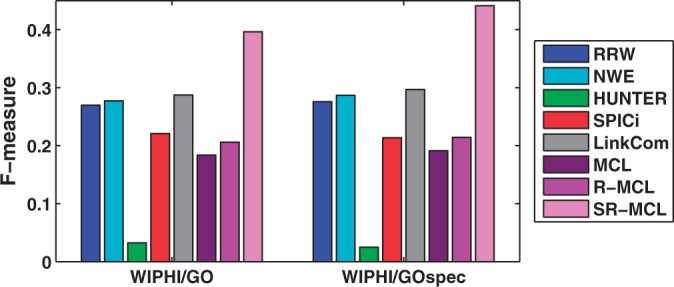
F-measure of each weighted network clustering algorithms on WI-PHI

**Fig. 4.**
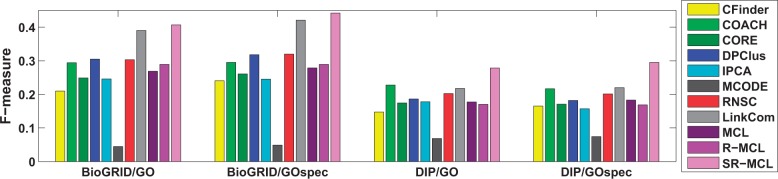
*F*-measure of each unweighted network clustering algorithms on BioGRID and DIP

SR-MCL has the highest precision among all algorithms except SPICi (See Supplementary Figs. 4 and 7), since SPICi produces less clusters that are only in dense subgraphs. With regard to recall, among all weighted network clustering algorithms, SR-MCL is the highest; and among all unweighted network clustering algorithms, SR-MCL and LinkCom are the highest on BioGRID, but RNSC is higher than SR-MCL on DIP (see Supplementary Figs. 5 and 9). This is simply because RNSC has much higher coverage (*>*4000) than SR-MCL (~2000), and therefore more GO-terms are identified. Nevertheless, as shown in Figures 3 and 4, SR-MCL has the highest *F*-measure among all existing algorithms on either unweighted or weighted networks. This is probably because SR-MCL has high precision due to the original design of R-MCL and the post-processing, and SR-MCL generally has high recall because it produces overlapped clusters after executing R-MCL multiple times with penalizing attractor nodes.

**Fig. 5.**
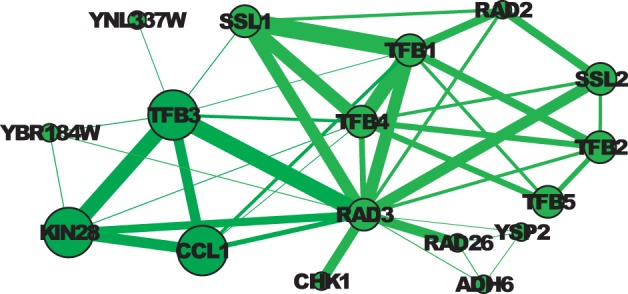
Two clusters matching GO:0005675 and its child term GO:0070985 in WI-PHI. The thickness of an edge represents the weight. The size of a node indicates the GO term annotation: The large nodes are annotated by GO:0070985 (and thus GO:0005675); the medium nodes are only annotated by GO:0005675; and the small nodes are are not annotated by either of these two terms. The color indicates the clustering result: All nodes form a cluster matching GO:0005675, and the dark green nodes form a cluster matching GO:0070985

Moreover, we have following observations about other algorithms: (i) HUNTER and MCODE's coverage are much smaller than other algorithms and their clusters are too large to identify small functional modules, resulting in extremely low (*<*5%) recall. Although MCODE's precision is higher than other algorithms except SR-MCL, its *F*-measure is lower than most other algorithms. SPICi also has very small coverage, but its clusters size is moderate, resulting in very high precision and low recall, and the F-measure is slightly lower than most of other algoirthms. (ii) CORE, DPClus, RNSC, MLC and R-MCL all have higher coverage and produce more and smaller clusters on the sparser network DIP than the denser network BioGRID. However, since the average size of gold standard are roughly equal in different networks and high-quality clusters are rarer in a sparser network, a functional modules identification algorithm should have lower coverage and produce less and roughly equal-size clusters on a sparser network. As a result, these algorithms have a relatively (compared with COACH and IPCA) lower F-measure on DIP than on BioGRID with regular-size GO terms. (iii) LinkCom clearly outperforms other algorithms except SR-MCL on BioGRID, but, LinkCom is just above average on DIP. This is because BioGRID is a denser network than DIP, and LinkCom can identify highly overlapped clusters in a denser network due to the line-graph transformation. (iv) RNSC, which is a hard clustering algorithms, surprisingly has average *F*-measure among all unweighted network clustering algorithms. Although RNSC's poor performance were previously reported by (Li *et al.*, 2010), after simply removing clusters containing two or less proteins, it can still have average performance, so it might be also interesting to extend RNSC into a soft clustering algorithm.

### 4.5 Identifying hierarchical annotations

In this section, we demonstrate that SR-MCL is capable of identifying hierarchical functional modules by showing two top cases. Each case contains two clusters matching a parent GO term and its child GO term.

In the first example, shown in Figure 5, the large nodes are all annotated by the child term ‘holo TFIIH complex’ (GO:0005675), and all of the medium nodes and the large nodes are annotated by the parent term ‘TFIIK complex’ (GO:0070985). SR-MCL produces two clusters matching these two terms. In an earlier iteration, a large cluster roughly matching the parent term with the attractor node RAD3 is produced. In a later iteration, after several nodes including RAD3 being penalized, a small cluster consisting of RAD3, TFB3, KIN28 and CCL1 is produced, where TFB3, KIN28 and CCL1 are the only three nodes annotated by the child term in WI-PHI.

**Fig. 6.**
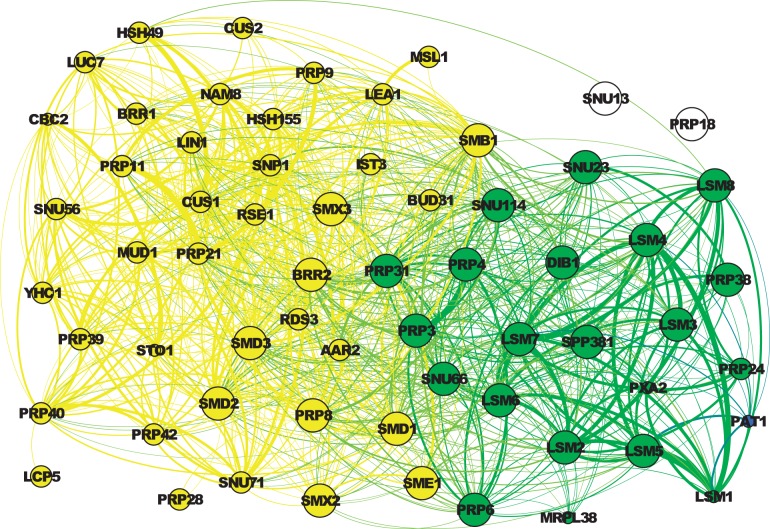
Two clusters matching GO:0030532 and its child term GO:0046540 in WI-PHI. The thickness of an edge represents the weight. The size of a node indicates the GO term annotation: The large nodes are annotated by GO:0046540 (and thus GO:0030532); the medium nodes are only annotated by GO:0030532; and the small nodes are are not annotated by either of these two terms. The color indicates the clustering result: The green nodes are the overlap of two clusters. The yellow nodes and green nodes form a cluster matching GO:0030532, and the blue node (PAT1) and green nodes form a cluster matching GO:0046540.

The second example is presented in Figure 6. The large nodes are all annotated by the child term ‘U4/U6 × U5 tri-snRNP complex’ (GO:0046540), and the medium nodes and the large nodes are all annotated by the parent term ‘small nuclear ribonucleoprotein complex’ (GO:0030532). SR-MCL produces two clusters matching these two terms: The cluster containing the green nodes and the blue node, named Cluster A, can roughly match the child term (GO:0046540), and the cluster containing the yellow nodes and the green nodes, named Cluster B, can roughly match the parent term (GO:0030532). Cluster A is produced in an earlier iteration with the attractor node LSM2; in a later iteration, as some other nodes in Cluster A were penalized in previous iterations, PRP6, which is roughly the center of nodes annotated by GO:0030532, becomes the attractor node of Cluster B.

## 5 CONCLUSIONS

In this article, we proposed a new approach for identifying functional modules in PPI networks—SR-MCL. We empirically found that SR-MCL outperforms a range of extant algorithms in terms of its accuracy in identifying functional modules. As part of future work, we are interested in auto-tuning some of the parameters of SR-MCL and adopting SR-MCL for TAP data (Gavin *et al.*, 2006; Krogan *et al.*, 2006).
